# Automated Force Volume Image Processing for Biological Samples

**DOI:** 10.1371/journal.pone.0018887

**Published:** 2011-04-29

**Authors:** Pavel Polyakov, Charles Soussen, Junbo Duan, Jérôme F. L. Duval, David Brie, Grégory Francius

**Affiliations:** 1 Laboratoire de Chimie Physique et Microbiologie pour l'Environnement, LCPME, UMR 7564, Nancy-Université, CNRS, Vandoeuvre lès Nancy, France; 2 Centre de Recherche en Automatique de Nancy, CRAN, UMR 7039, Nancy-Université, CNRS, Vandoeuvre lès Nancy, France; 3 Laboratoire Environnement et Minéralurgie, LEM, UMR 7569, Nancy-Université, CNRS, Vandoeuvre lès Nancy, France; Swiss Federal Institute of Technology Zurich, Switzerland

## Abstract

Atomic force microscopy (AFM) has now become a powerful technique for investigating on a molecular level, surface forces, nanomechanical properties of deformable particles, biomolecular interactions, kinetics, and dynamic processes. This paper specifically focuses on the analysis of AFM force curves collected on biological systems, in particular, bacteria. The goal is to provide fully automated tools to achieve theoretical interpretation of force curves on the basis of adequate, available physical models. In this respect, we propose two algorithms, one for the processing of approach force curves and another for the quantitative analysis of retraction force curves. In the former, electrostatic interactions prior to contact between AFM probe and bacterium are accounted for and mechanical interactions operating after contact are described in terms of Hertz-Hooke formalism. Retraction force curves are analyzed on the basis of the *Freely Jointed Chain* model. For both algorithms, the quantitative reconstruction of force curves is based on the robust detection of critical points (jumps, changes of slope or changes of curvature) which mark the transitions between the various relevant interactions taking place between the AFM tip and the studied sample during approach and retraction. Once the key regions of separation distance and indentation are detected, the physical parameters describing the relevant interactions operating in these regions are extracted making use of regression procedure for fitting experiments to theory. The flexibility, accuracy and strength of the algorithms are illustrated with the processing of two force-volume images, which collect a large set of approach and retraction curves measured on a single biological surface. For each force-volume image, several maps are generated, representing the spatial distribution of the searched physical parameters as estimated for each pixel of the force-volume image.

## Introduction

The physico-chemical characterization of biological materials in general, and bacteria in particular, is an important challenge in domains as diverse as biology, microbiology, pharmaceutic and environmental industry, as well as in the field of clinical medicine. Determination of physico-chemical properties of bacteria in terms of electrostatic charge or elasticity is of fundamental relevance *e.g.* for understanding bacterial adhesion and infection processes. In addition, several analyses have now evidenced that external structures at the outer periphery of bacteria, like biopolymers or proteins, characterized by different physico-chemical properties, play distinct roles in numerous physical and biological interfacial processes, *e.g.* plasmid transfer through conjugation [1], adherence to materials or host cell surfaces [2], cell-cell interactions [3], biofilm formation [4], [5], [6], [7], [8], mobility [9], [10], [11] and pathogenicity [12], [13], [14], [15].

Atomic force microscopy (AFM) emerged within the last decade into a powerful tool for various physical and biological applications [16], [17]. It can operate under wet or physiological conditions [18], [19] with sub-nanometric spatial resolution. AFM force spectroscopy now allows probing of mechanical properties of soft biological samples [20], [21], [22], [23], [24] and measurement of inter- and intramolecular interactions between biomolecules, thus providing new insights into the molecular bases of macromolecular elasticity [25], [26], protein folding [27], and receptor-ligand interactions [28]. AFM is now regarded as a very suitable technique for investigating processes connected to molecular recognition, and for providing valuable information at a molecular scale on the dynamics of individual ligands and receptors on biosurfaces [29] *via* the analysis of retraction force curves. Mechanical properties of soft samples can be evaluated upon appropriate interpretation of approach force curves, so-called nanoindentation analysis [30], [31], [32]. According to the latter, viscoelastic properties of living cells may be recovered [33], *e.g.* elasticity of human cells [34], [35], [36], [37], bacteria [38], [39], [40], or soft gels [41], [42]. In addition, other physico-chemical parameters like charge density or turgor pressure of bacteria covered by specific proteins, polysaccharides or lipopolysaccharides, are now accessible by AFM [43]. In view of the recent numerous and impressive developments AFM technique has undergone, we may state that it is now a necessary tool for understanding a large spectrum of biochemical and biophysical signals which are of utmost importance in clinical medicine [44], [45], [46], life science [47], [48], [49], [50], environmental science [51], and cosmetic industry [52]. For the sake of further illustration, previous works in nanomedicine have demonstrated that cancer, tumor and stem cell biology are regulated by mechanical properties of cells [53], [54], [55], [56] and some diseases can now be diagnosed with use of AFM [57], [58], [59], [60].

The analysis of complex, heterogeneous biological systems can be performed through force-volume imaging (FVI) in which a set of force curves are being recorded on a spatial grid defined on a given sample surface. Analysis of such FVI aims at providing *in fine* a mapping, *i.e.* a spatial distribution of the relevant physical parameters pertaining to the biological sample. Because of the large amount of force curves necessary to generate a FVI, there is a critical need to develop robust computational methods to achieve:

- accurate determination of the physical parameters of interest all over the biological sample surface;
- fast and automated processing of each force curve in the whole FVI.

This is essentially the purpose of this paper. In details, we are interested in determining the electrostatic and mechanical properties of biological particles like bacteria. This is achieved upon quantitative analysis of the approach curve between sample and AFM tip prior to and after contact. For that purpose, the force curves are modeled by a piecewise parametric model (electrostatic interaction, Hertz interaction, Hooke interaction) allowing for the concomitant estimation of the spatial range where electrostatic interactions are operative, and the evaluation of the Young modulus and the spring constant of the bacteria. This clearly constitutes a major contribution because the currently available computational methods [61], [62], [63], [64], [65] essentially allow for the estimation of physical parameters pertaining to mechanical interactions only. We also address the automated analysis of biopolymer uncoiling in the course of retraction of the tip from the sample surface (retraction force curves). The issue is then to perform an accurate interpretation of the non-monotonous retraction force curves by means of *Worm Like Chain* (WLC) or *Freely Jointed Chain* (FJC) models. To the best of our knowledge, analysis of retraction force curves according to these models is commonly done *via* a manual, *ad hoc* and time-consuming specification of the various intervals in spatial separation between AFM tip and sample. We propose here a fully automated method for fitting retraction force curves on the basis of the aforementioned models. Similar to the analysis of the approach curves, the method consists in a piecewise parametric modeling of the force curves recorded upon retraction of the tip from the investigated sample.

When fitting a series of physical models to reconstruct the various interaction regimes pictured by a given force curve, a key issue is the accurate determination of the various zones where one type of interaction is operating, or equivalently the evaluation of the critical points (also called change points in statistical signal processing) marking the transition between two interactions of different natures, *e.g.* electrostatic and mechanical interactions upon gradual approach of the AFM tip towards the sample. Another difficulty is that the parametric models necessary to recover the approach force curves do not apply within the same regime of tip-to-sample separation: the electrostatic model indeed essentially depends on the probe-to-sample separation distance while the contact models (Hertz's and Hooke's theories) involve the so-called deformation depth or indentation of the sample. A mandatory prerequisite for appropriate definition of the latter is the accurate localization of the ‘contact point’ [41], [66]. Many studies have already stressed this important issue, and in particular highlighted that determination of the contact point is a tricky operation for deformable biological samples [61], [62], [63], [64], [65]. This is also true for situations as simple as those requiring the sole application of Hertz model where neither electrostatic interaction nor linear Hooke mechanical deformations are considered [67], [68]. From a numerical point of view, finding critical points requires to solve a *discrete* optimization problem as the indices of the critical data points must be estimated. This is far from being an easy task within a limited computation time. Indeed, a force curve typically includes thousands of data points and exhaustive discrete search algorithms are known to be numerically time-consuming when the search domain is large. Once the critical points are found, fitting of the experimental data to a given physical model within the required spatial range can be carried out by means of standard local continuous optimization algorithms leading to the estimation of the searched key parameters. For a limited number of unknown parameters, running continuous optimization algorithms is a rather fast process, although there can be issues depending on the degree of sophistication of the models (local minimizers, flat cost function) [52].

The paper is organized as follows. First, we outline the physical models used for reconstructing the approach and retraction force curves. The physical models correspond to the different interactions (electrostatic, mechanical and adhesion forces) that take place between biological samples and AFM tip. Then, the computational method employed for extracting the physical parameters of interest is thoroughly described. To illustrate the performance of our analysis and perform a mapping of bacterial cell physical properties (*e.g.* elasticity, adhesion), we selected two bacterial models. We studied respectively *Escherichia coli* K12 mutant devoid of any cell surface appendage [43], and performed single-molecule force spectroscopy (SMFS) experiments on *Pseudomonas fluorescens* because of its exopolysaccharide production. In the last part of this paper, we thoroughly discuss the results and comment on the benefits of our computational method for the analysis of force-volume images.

## Materials and Methods

The quantitative analysis of a given force curve requires specific models describing the different physical interactions occurring during approach and retraction. In particular, we focus on the electrostatic interaction between *E. coli* and AFM tip during the approach of the tip, and further analyze the subsequent deformation of the sample after contact with the tip. For retraction curves, we use FJC-based models to describe the stretching of *Pseudomonas fluorescens* bacterial polysaccharides [21], [69], [70]. In this section, we introduce the studied bacteria and describe the sample preparation procedure. Next, we detail the conditions of data acquisition with the AFM instrument and then we describe models and data processing.

### Bacterial culture and sample preparation

The first bacterial model used in this study is a gram negative *Escherichia coli* K-12 mutant called (E2152) kindly provided by the Institut Pasteur of Paris. This bacterium was constructed from *E. coli* MG1655 (*E. coli* genetic stock center CGSC#6300) by transformation and λ red linear DNA gene inactivation method followed by P1*vir* transduction into a fresh *E. coli* background when possible. E2152 is characterized by a simple gram negative bacterial cell wall that does not exhibit any biopolymers or external structures at its outer periphery [43]. To extract relevant electrostatic and mechanical properties, we analyze approach force curves collected between the bacterium and standard silicon nitride AFM tip.

Secondly, we analyze retraction force curves pertaining to the uncoiling of exobiopolymers (probably glycogene) from *Pseudomonas fluorescens* using single-molecule force spectroscopy (SMFS). *P. fluorescens* is a common bacterium present in drinking water distribution network [71] and can form biofilms [72] and produce exopolymers [73]. In this analysis, AFM tips are functionalized with *Concanavalin A* lectin in order to detect mannosyl and glucosyl residues present in the bacterial exopolymers (EPS) [74], [75]. Specific binding and deactivation of ConA tips to glucosyl and mannosyl residues was verified on a surface coated with glucose (see Fig. S4a and S4b). Before each single-molecule force spectroscopy (SMFS) experiment, it was systematically verified that ConA tips did not bind specifically to the PEI-coated glass where bacteria were immobilized prior to experiment (see Fig. S5).

### AFM measurements and preparation of experiments

AFM images and force-distance curves were recorded using an MFP3D-BIO instrument (Asylum Research Technology, Atomic Force F&E GmbH, Mannheim, Germany). Silicon nitride cantilevers of conical shape were purchased from Veeco (MLCT-AUNM, Veeco Instruments SAS, Dourdan, France) and their spring constants were determined using the thermal calibration method [76], providing values of ∼10.4±1.7 pN/nm. Prior to each experiment, the geometry of the tip was systematically controlled using a commercial grid for 3-D visualization (TGT1, NT-MTD Compagny, Moscow, Russia) and curvature of the tip in its extremity was found to lie in the range ∼20 to 50 nm. FVI experiments were performed adopting the value 1000 nm/s for the approach/removal speed of the tip from/toward the sample surface. Upon successive approach and retract force measurements done at a given location of the surface with such a tip displacement velocity, similar force curves were obtained, thereby indicating that we are proceeding under conditions where the surface has enough time to fully relax before data acquisition. This also fully justifies the use of static physical models as adopted here for data analysis. Experiments were carried out in 1 mM potassium nitrate solution at pH ∼6.6 and room temperature. Note that only one force curve were recorded by FVI-pixel to achieve a reasonable acquisition time of about 20 to 30 minutes for whole FVI. In our work, only *z* piezo is closed looped, the lateral resolution in *x* and *y* is about 0.50 nm and the vertical resolution about 0.25 nm. The noise in the tip to sample distance is lower than 0.06 nm for the height and lower than 0.02 nm for the deflection.

Because previous studies pointed out the possible removal and/or shortening of bacterial appendages upon sample centrifugation [77], bacterial cultures were used without any particular conditioning regarding AFM experiments. Cells were electrostatically immobilized onto polyethyleneimine (PEI)-coated glass slides according to a procedure detailed elsewhere [78]. Such method avoids the necessity to resort to chemical binders between substrate and bacterial sample, thus minimizing any chemical modification of bacterial cell wall/surface organization.

Glass slides were freshly prepared upon immersion in 0.2% PEI solution for 30 minutes, extensively rinsed with Milli-Q water, dried with nitrogen and stored in a sterile Petri dish. One mL of bacterial culture (OD_600nm_ ∼0.5–0.6) was directly deposited onto the PEI-coated glass slide for 20 minutes and then the bacteria-coated surface was extensively rinsed 3 times with Milli-Q water. Following this step, the sample was immediately transferred into the AFM liquid cell with addition of 2 ml of KNO_3_ solution of adjusted concentration and pH∼6.6 for imaging and nanomechanical analysis. Single Molecule Force experiments were performed with 2 ml of standard buffer solution (40 mM maleic acid, 60 mM TRIS, 2 mM CaCl_2_ and 2 mM MnCl_2_ at pH 5) and AFM tips were functionalized with *Concanavalin A* (ConA) [74], [75], [79].

### Physical models relevant for the approach curves

In situations where AFM tip and bacterium are gradually brought together, electrostatic interactions between tip and bacterium first take place, followed by mechanical contact and deformation of the cell envelope as a result of the compression exerted by the tip. The underlying force is not measured directly, but evaluated according to Hooke's law:
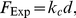
(1)where *F*_Exp_ is the experimental force measured by AFM, *d* is the deflection of the cantilever and *k_c_* the known spring constant of the cantilever. Three regimes in the measured approach force curve may be considered for analysis and in turn allow estimation of (i) the volume charge density within the soft envelope of the bacterial cell and the Debye layer thickness (κ^-1^) that pertains to the typical spatial range where electrostatic interactions between sample and tip are operative, (ii) the Young modulus (*E*) which reflects cell surface elasticity, and (iii) the bacterial spring constant (*k*_cell_) that is related to the inner turgor pressure (*P*_0_) of the cell.

First, upon approach of the (silicon nitride) AFM tip toward the bacterium, both being generally negatively charged [43], [80], repulsive electrostatic double layer force *F*_Elec_ needs to be considered. For sufficiently large separation distances where the electric double layers developed around the charged sample and tip weakly overlap, *F*_Elec_ may be expressed within the Debye-Hückel approximation as detailed by Ohshima [81]. The expression for *F*_Elec_ may then always be written in the form [82]:
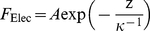
(2)where *z* is the probe tip to cell surface separation distance, *κ* ^−1^ the Debye layer thickness and the scalar prefactor 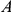 is a function of the dielectric constant of the medium *ε*_o_ *ε*_r_, the surface charge density *σ* of the hard probe tip, the thickness *h* and volume charge density *ρ* of the soft cell envelope. More detailed theory is required to determine the exact mathematical dependence of *A* on the searched physicochemical properties of the cell envelope [84], those of the tip being generally known from independent AFM or electrokinetic measurements [83]. It is beyond the scope of the current paper to provide these expressions that are also depending on tip geometry. For the sake of illustration, in case of electrostatic interaction between a planar bacterial interphase of thickness *h* and a hard AFM spherical tip apex of radius *R*, we obtain 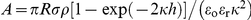, which is derived from the potential energy expressed by eq 24 in [84] taken in the limit of weak electric double layer overlap. The latter relation is further valid providing that curvature effects on electrostatic field distribution are negligible and radius of the tip is much lower than bacterial radius. For situations where curvature effects must be taken into account, numerical analysis of governing electrostatic equations is necessarily required and the reader is referred to the recent general theory by Duval et al. for this purpose [84]. For the sake of generality, we reason in the following on the basis of the general expression given by eq (2) and focus on the automated determination of the key prefactor *A* that contains all searched information pertaining to the electrostatic features of the investigated sample. The quantity *κ* ^−1^ is depending on the concentration of electrolyte in solution according to the expression:
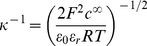
(3)with 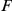 the Faraday constant, *R* the gas constant, 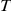 the temperature, ε_0_ the dielectric permittivity of vacuum, ε_r_ the relative dielectric permittivity of the aqueous medium, *c* ^∞^ the bulk concentration of the here-considered monovalent electrolyte. The automated analysis described below for obtaining the prefactor *A* also allows evaluation of *κ* ^−1^ [84]. The consistency of the analysis may then be checked upon comparison of the so-determined *κ* ^−1^ with the theoretical prediction given by eq (3). It is emphasized that for repulsive interactions the prefactor *A* is positive while for attractive interactions it changes sign. Situations where *A*<0 may be encountered for interactions of bacteria with chemically modified AFM tips carrying a positive surface charge [83].

Once the AFM tip and the cell envelope are in contact, mechanical deformation of the cell envelope takes place. In details, one may distinguish non-linear and linear deformation of the cell envelope upon compression by the AFM tip. The bacterial Young modulus *E* is obtained from quantitative interpretation of the non-linear regime that follows the electrostatic part detailed above. The corresponding interaction force, denoted as *F*_Hertz_, is a function of the so-called indentation *δ i.e.* the deformation of the bacterial wall. We apply the Hertz model [85] which is relevant for the deformation of a soft planar interface under action of a tip of conical geometry:
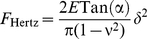
(4)where *ν* is the Poisson coefficient (0.5 in case of incompressible medium) and *α* is the semi-top angle of the tip (35° – value given by manufacturer). The bacterial spring constant value *k*_cell_ is related to the slope of the linear part of the force *versus* indentation curve that follows the aforementioned non-linear regime [86]:
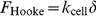
(5)The automated analysis described below allows for estimation of the prefactors 
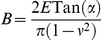
 and *k*_cell._ The obtained value of *k*_cell_ is related to the value of the turgor pressure of the cell as detailed in Fig. S1.

### Physical models relevant for the retraction curves

In retraction experiments, biomacromolecules located on the surface of the biological sample are stretched upon removal of the chemically modified AFM tip away from the surface. The obtained force *versus* distance curves are then analyzed according to FJC models [87], [88], [89]. This choice is justified because the studied bacterial model (*P. fluorescens*) produces exopolysaccharides, as suggested by recent FTIR measurements (data not shown, paper in preparation). In addition, FJC models are the most frequently used in the literature [21], [70], [74], [89], [90] to describe the polysaccharide extension behavior because polysaccharide monomers can be regarded as independent rotating segments unlike protein constituents for which WLC models are more suitable [91] (see further details in Fig. S6). More details are given in the ‘Results and Discussion’ section for the preparation of the bacterial strains investigated here.

Within the framework of the FJC model, it is assumed that the macromolecule consists of rigid segments connected through flexible joints. The extension 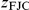 of the macromolecule may then be expressed as a function of the pulling force *F via* the expression [87], [88]:
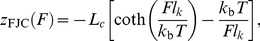
(6)where the Kuhn length *l_k_* is a direct measure of the chain stiffness, 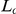 is the total contour length of the macromolecule and 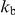 is the Boltzmann constant. It should be noted that the number of monomers *N* in the biomacromolecule is simply related to *L_c_* and *l_k_* according to *N* = *L_c_*/*l_k_*.

For the sake of simplicity and conciseness, we choose to mainly consider the FJC model within the algorithm described below. The flexibility of the algorithm to generate data fitting according to the FJC+ model will be discussed in the ‘Results and Discussion’ section. In addition, the first region in the retraction curve (*i.e* the first ∼100 nm) was not taken into account for retrieving information on macromolecule conformation, in line with strategy classically adopted in the literature [90], [91].

### Algorithms

For both approach and retraction cases, the models introduced above are *piecewise models* for force curves, *i.e.* they are strictly applicable within well-defined (but unknown *a priori*) regions of separation distance *z*, indentation *δ* or macromolecular extension. In the approach case, the electrostatic regime applies until the contact point is reached. Then, after contact, the Hertz and Hooke regimes successively take place upon gradual compression of the bacterial cell by the tip. In the retraction case, expressions derived from the FJC formalism can be viewed as a piecewise equation: the unfolding of molecules leads indeed to a succession of 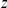-intervals where the FJC model holds for quantitative interpretation.

**Figure 1:**
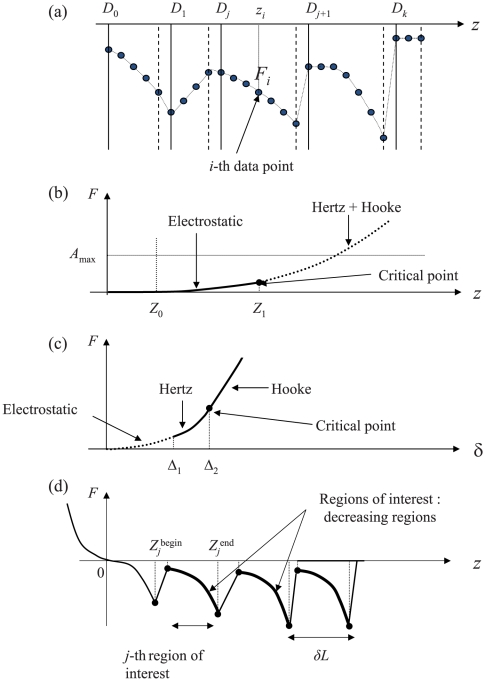
Main notations and schematic illustration for the three steps of the proposed algorithm. (a) Segmentation of a force curve (first step). The data points (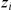, 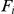), *i = 1*,…,*n* are partitioned into *k+1* contiguous intervals 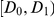, 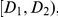
…,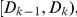 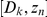 with 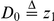 and 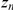 the minimal and maximal 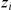 values. The plain and dashed vertical lines represent the left and right bounds of the segmented intervals. The experimental data 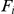 are smoothed on each interval (piecewise smoothing). (b) Detection of the electrostatic region in the 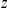-domain (second step) and fitting to the electrostatic model (third step). The critical points 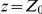 and 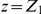 are the edges of electrostatic region in the 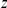-domain. *A*_max_ is an upper bound for the exponential prefactor *A*. It is optional but useful for improving the detection of the edge 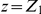 by forbidding 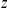-values that lead to unrealistic values of *A*. (c) Segmentation and fitting in the δ-domain (third step). The critical points 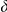 = 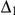 and 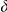 = 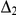 correspond to the beginning of the Hertzian and Hooke regimes, respectively. (d) Retraction curve: the *j*–th region of interest is the *j*–th decreasing interval 
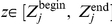
. The search for these regions is done from the outputs of the segmentation algorithm (second step), then the data are fitted to the FJC model within each region of interest (third step).

A key prerequisite for the quantitative analysis of a force curve (*i.e.* for the estimation of the relevant physical parameters) is the accurate identification of the various intervals where each model may be applied, and corresponding regression procedures performed. These intervals, or regions, are illustrated on Figures 1b, 1c and 1d in the case of approach and retraction curves, respectively. In the following, they will be referred to as *regions of interest*. Their identification relies on a *force curve segmentation* procedure allowing for the detection of a set of *critical points* (or discontinuity points: jumps, change of slope or change of curvature). The proposed algorithm decomposes the problem into three steps:

1. Force curve segmentation;
2. Detection of the regions of interest where modeling may be carried out;
3. Fitting of the data to the physical models in each region.

The first step is common to the processing of approach and retraction curves. It will be described first. On the contrary, the two other steps differ because they rely on distinct physical models. For readability reasons, we will describe the processing steps 2 and 3 for the approach force curves first, and then for the retraction case.

#### Force curve segmentation

The force curve segmentation procedure consists in the following two tasks:

- Detection of the discontinuity points in the given measured signal;
- Piecewise-smooth approximation of the signal.

The notations are defined in Figure 1a. *z_i_* and *F_i_* stand for the *i*-th experimental measurement where *i* is increasing with increasing acquisition time. The 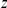-axis is defined in such a way that the 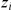 values are increasing with increasing 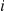: the probe-to-sample contact corresponds to the largest 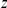-value in the approach curve and to the lowest 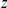-value for the retraction curve. All force curve data points have been pre-processed beforehand to re-order the z values so that the z*_i_* are systematically increasing with increasing 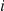. Note that in the approach case, the electrostatic force *F*_Elec_ is now increasing with *z*, thus eq (2) rereads 
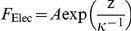
.

Let *D*_1_, …, *D_k_* refer to the (unknown) discontinuity points, leading to a series of contiguous intervals 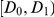, 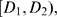
…,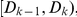 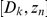 with 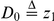 and 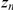 the minimal and maximal 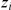 values. Each interval is left-closed and right-opened (except for the last one) so that their union provides the whole interval 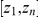 (see Figure 1a). On each interval, the experimental data are smoothed in the following way. The force measurements 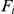 corresponding to 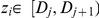, are approximated by a polynomial 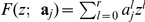. The approximation is done in the least-squares sense by minimizing the squared error
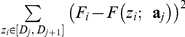
 with respect to 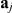.

**Figure 2:**
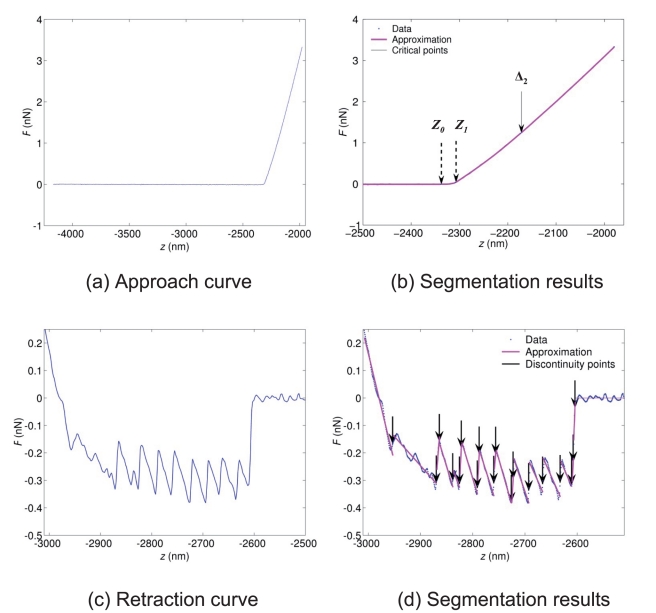
Force curve segmentation. The approach and retraction curves correspond to the marked pixel in the *E. coli* cell image shown in Figure 4c. (a) Approach curve: experimental data. The curve is preprocessed by subtraction of an affine baseline. (b) Segmentation results with 6 discontinuity points. In the interval *z* ∈ [−2500, −2000] nm, only three discontinuity points are present. Both points represented with dashed arrows are the edges 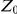 and 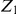 of the electrostatic region. The next discontinuity point (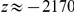 nm) marked by a plain arrow is used as initial estimation of the transition between Hertz and Hooke regimes. (c–d) Retraction curve. The polynomial degree is set to 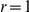 leading to a piecewise affine approximation of the original signal. The threshold value 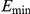 for the mean squared error is set to the empirical noise variance, and 20 discontinuities at most are detected. For the displayed retraction curve, 20 iterations were performed leading to 20 discontinuities.

The segmentation algorithm is inspired by the Orthogonal Least Squares algorithm [92]. This procedure gradually approximates a given signal upon selection of an increasing number of elements from a predefined set of elementary signals. Basically, the segmentation algorithm searches for 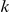 discontinuity points for which the sum of the squared errors 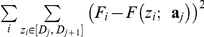 is minimal. It is based on the iterative addition of one element into the list of discontinuity positions. In the first iteration, this list is empty. The algorithm then sequentially includes a new discontinuity point and updates the piecewise-smooth approximation until 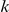 discontinuities are found. An important issue is the setting of the number of discontinuity points. A first possibility is to define a maximal number of points upon visual inspection of the experimental signal and simple count of the number of discontinuity points. An alternative and more automated procedure consists in setting a threshold value 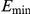 for the mean squared error, *i.e.* the average of the approximation error 
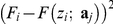
 for all 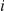 and intervals 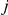. If the mean squared error is lower than 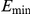, then the algorithm is stopped. The reader is referred to a more formal and detailed description of the segmentation algorithm in a technical report reported in [93]. In addition, more details are given in the ‘Results and discussion’ section on the setting of the number of discontinuity points. The segmentation algorithm is illustrated in Figure 2 for the approach and retraction cases.

#### Fitting of the approach curves

In this section, we describe in details the two remaining steps for quantitative analysis of an approach curve. Let us first introduce formal notations for the critical points marking the onset and the end of the electrostatic interaction in the 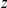-domain. To distinguish them with the data measurements 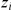, we use capital letters 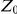 and 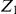 (see Figure 1b). We define the point 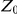 as the 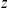-value below which instrumental limitation renders impossible any accurate measurement of interaction force. 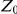 can be seen as a ‘virtual pre-contact point’. It does not correspond to a physical change point nor to a real contact between the AFM tip and the sample. However, it is necessary to consider such arbitrary position to define the indentation 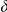. The 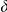-values are defined according to 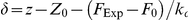 where 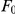 is the force value at 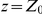 (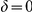 for 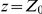). The next critical point 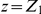 corresponds to the transition from the electrostatic to the Hertz regimes (Figure 1b). Because the non-linear and linear mechanical deformations of the cell wall are defined in the indentation domain, the transition from the Hertz to the Hooke regime is defined accordingly using the quantity 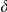. This position, denoted as 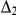, will be estimated in the regression procedure described below. In brief, for 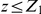, the electrostatic regime holds; the contact point (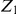) has not been reached; for 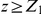 and 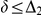, the Hertz regime holds; for 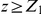 and 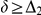, the Hooke regime holds.

In the second step of the algorithm, the regions of interest 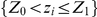 (electrostatic regime) and 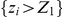 (Hertz and Hooke regimes) are detected based on the segmentation results. The discontinuity points provided by the segmentation algorithm are used in the following manner. Let 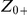 be the first data point satisfying the condition 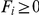 for all 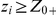. 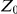 and 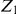 are then defined as the two *consecutive* discontinuity points 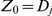 and 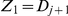 surrounding 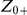 (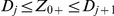). It should be noted that in the noise free case 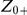 would correspond to the first data point, thus leading to incorrect detection of the electrostatic region. However, as we are processing experimental data, it is very unlikely that this situation happens. At least, it has never been observed for all the force curves processed in this work.

The detection of the electrostatic region is illustrated on Figure 2b where the critical points 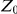 and 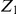 are explicitly indicated. Also, the following discontinuity point 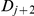 is used as an initial estimation of the edge between the Hertz and Hooke regions in the 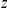-domain: 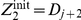. The final estimation of this edge position (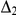) is done in the 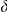-domain.

Once both electrostatic and contact regions are clearly identified, the remaining task (third step) consists in a fit of the experimental data to the appropriate physical models. The unknown parameters are estimated in the least-squares sense, *i.e.* we minimize the cost function defined as the sum of the squared errors between experimental data and their approximation from the parametric model. We now define two cost functions corresponding to the electrostatic and mechanical interactions. In each case, we detail the imposed constraints on the parameter values. Because we use a local gradient-based algorithm whose behavior can depend on the setting of the initial parameter values, we also detail the initialization step and propose heuristic rules for each model.

In the 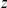-domain, the electrostatic model 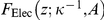 holds for 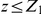 (see eq (2)). The fitting of the experimental data 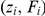 for 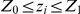
is formulated as the minimization of the cost function:
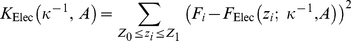
(7)under the constraints 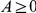 (repulsive interactions) and 
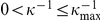
 (see ‘Results & Discussion’ section for practical setting of 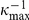). The dependence of 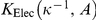 on 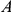 is quadratic because 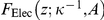 is linear in *A* (see eq (2)). Therefore, a closed-form expression for 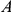 can be derived from 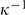 and the minimization of eq (7) then reduces to a 1D minimization problem 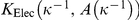.

In the 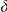-domain, the contact (Hertz and Hooke interactions) model reads:
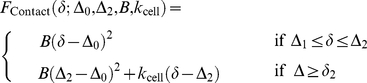
(8)where the centering around 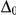 is introduced so as to take into account an unknown ‘reference/virtual’ pre-contact point. Equations (8) ensure that the force is continuous at 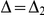 *i.e*. there is no discontinuity between the Hertz and Hooke regimes. Similarly, 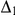 is set to 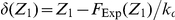 in order to impose that the force curve model is continuous at 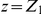. The estimation of the four parameters 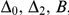
and 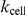 is done by computing the indentation 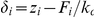 for all 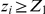 and by minimizing 
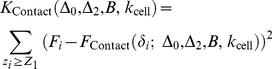
(9)under the constraints 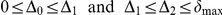 where 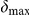 stands for the maximum of all 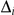-values. Since the dependence of 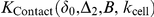 on 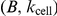 is quadratic, the minimization of eq (9) simplifies into a 2D problem, as 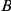 and 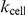 may be expressed as a function of 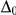 and 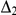 according to a closed-form relationship.

The cost functions 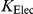 and 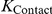 may contain ‘flat valleys’. Also, there may be several local minimizers. This makes the optimization algorithm sensitive to the choice of the initial parameter values (as already mentioned for the electrostatic fitting problem [63]). Thus, it is critical to initialize the algorithm with physically realistic values. Here, we detail the initialization of each parameter.

- For the electrostatic model, the initial value of 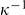 is set having in mind the relationship 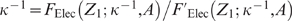, where 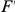 stands for the derivative of 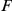 with respect to 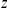. In practice, we use the smoothed polynomial signal obtained as output of the segmentation algorithm and we compute the ratio between the polynomial value and its derivative at 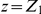. As previously mentioned, 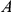 directly derives from 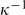 using the closed form expression that relates the two parameters.
- During the segmentation procedure, a rough estimation for the edge between the Hertzian and Hooke regions, 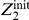, is computed. This estimation leads to an initial evaluation of 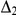: 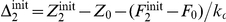 with 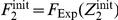. We set the initial value for 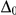 to 0.

**Figure 3:**
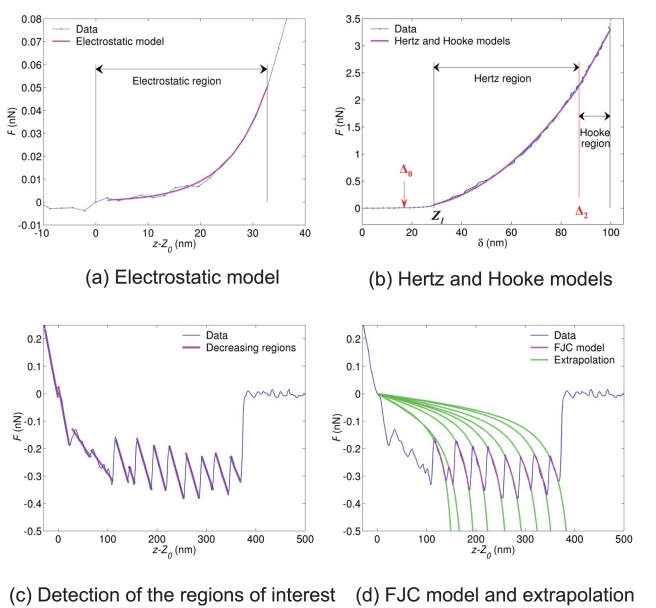
Detection of the regions of interest from the segmentation results and fitting to the physical models. (a) Approach curve: both vertical lines correspond to the beginning (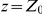) and end (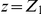) of the electrostatic regime. (b) Approach curve, Hertz and Hooke regimes. The red vertical line and red arrow correspond to the positions 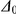 and 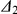 which are found by the optimization algorithm. The black line in between marks the 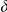-value 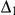 for 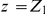 (beginning of Hertzian regime). (c) Retraction curve: detection of the regions of interest (decreasing regions of the piecewise affine signal). (d) Fitting of the data to the FJC model in each region of interest. No fitting is performed in the first 100 nm after the contact point 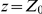. For each region, the extrapolation of the FJC model outside the current region of interest is shown in green.

An illustration of the electrostatic fitting is displayed in Figure 3a while the fitting results pertaining to the contact part of the force curve, including the estimation of the 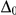 and 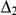 positions, are shown in Figure 3b.

Fitting of the retraction curves. Similar to the approach case, we now detail for the retraction curve the two remaining steps of the fitting algorithm given the outputs 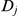 of the segmentation procedure.

There are as many regions of interest as number of segments in the freely jointed chain. Contrary to the approach model, the regions of interest 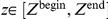 are not contiguous: two ‘consecutive’ regions may be separated as indicated in Figure 1d. These regions are denoted as 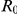, 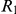, … and generally differ from the intervals 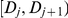 provided by the segmentation algorithm. We now detail their detection according to heuristic rules. Because the FJC curve (seen as a function of *z*: 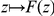) decreases in all intervals of interest, we basically consider the intervals 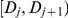 where the polynomial approximation 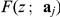 is a decreasing function. The FJC model (eq (6)) then applies between 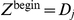 and 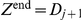. The detection of the regions of interest is illustrated in Figure 3c together with the piecewise smoothing (piecewise affine smoothing, obtained with polynomial degree 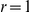). The contact point 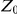 is defined from the corresponding approach curve as detailed above. When the polynomial degree is set to 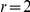, 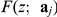 may not be monotonous within the interval 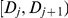. For such situations, we split the interval into two sub-intervals where 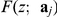 is monotonous, and solely consider the sub-interval 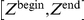 where the quadratic polynomial is a decreasing function. An example of this kind of situation is provided in Fig. S2 where the segmentation is performed with second order polynomials. Finally, intervals 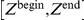 with 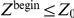 and intervals containing very few (typically, less than 5) data points are not considered.

For each region of interest 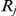, the FJC parameters are the contour length 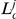 and the Kuhn length 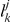. Their recovery is carried out by fitting the data 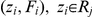 to the FJC model. The least-squares formulation leads to the minimization of
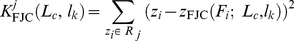
(10)under the constraints 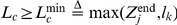 and 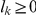 where 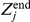 denotes the upper bound of the *j*-th region of interest. The minimization of eq (10) is a 2D optimization problem quadratic in 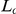. It thus simplifies into the 1D problem 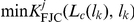 subject to 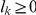, where for a given 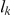, 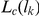 stands for the minimizer of 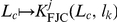 subject to the constraint 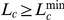 which has a closed-form expression.

We noted that the 1D cost function 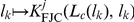 exhibits large variations for small values of 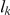 (convex valleys) and becomes very flat for larger 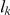. When setting an initial solution of the same order of magnitude as the 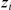-values (*e.g.* 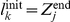), 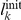 is found to lay within the flat valley of the cost function, and the local optimization algorithm then often fails to find the global minimizer of the cost function. In practice, we rather set the initial condition to 
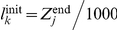
 to ensure that 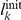 lays in the non flat convex valley, and close enough to the global minimizer of 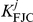. In the ‘Results & Discussion’ section, we discuss the issues relative to fitting to the extended FJC+ model.

#### Software for the processing of force-volume images

The software dedicated to the automatic processing of a FVI has been implemented in Matlab and uses the Optimization toolbox to perform the local optimization tasks described above. The processing of a FVI consists in applying the fitting procedure to each force curve, *i.e.* for any pixel in the FVI. The force curves are processed sequentially, thus providing as many outputs (physical parameters) as there are pixels. In the case of approach curves, each physical parameter can then be displayed on a 2D map. For retraction curves, the number of regions of interest differs from one curve to another. A global (but complex) visualization of each parameter would take the form of a 3D image since there are many 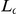 and 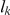 values per pixel. For simplicity reasons, in the ‘Results section’, we rather choose to display the global histograms of the 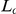 and 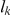 parameters obtained for all pixels, and we display two 2D maps representing the adhesion force corresponding to the last jump *F*(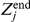) and the last rupture distance 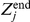.

The computational time of the algorithm has been evaluated upon processing of the experimental data with a standard DELL Latitude 810 computer (CPU 2GHz and 2 Go RAM). For force curves composed of around 2700 data points, the running time is less than 2 seconds for the approach curves, and it is about 25 seconds (degree *r* = 1, search for 20 discontinuities) and 45 seconds (degree *r* = 2, 20 discontinuities) for retraction curves. In the algorithm, the segmentation step is most time consuming, the other two steps being very fast (they are almost instantaneous for approach curves and take less than 5 seconds in the retraction case). The speed of the segmentation procedure depends on two crucial factors, namely the number of iterations and the threshold value 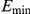 pertaining to the mean squared error. Overall, the processing of a force-volume image corresponding to a grid of size 32×32 pixels requires 22 minutes and 4 hours in the approach and retraction cases, respectively (with a polynomial degree set to *r* = 1). It should be noted that the above time scales could be drastically reduced with an implementation of the algorithm in a compiled language (*e.g*. in C). An important point is that the algorithm is very well suited for the processing of force curves having a large number of data points (typically, the experimental force curves include from 2500 to 3000 data points). There is no strong memory requirement since force curves are processed sequentially, *i.e.* in an independent manner. It is emphasized that we always guide the algorithm towards physically realistic solutions. As an example, the *κ* and *A* parameters in the electrostatic model are imposed to be non-negative. In the algorithm, two parameters are introduced to express that for approach curves, the force value at the ‘contact point’ should not be larger than *A*_max_ (empirically determined) which serves as a constraint (upper bound) for fit of data in the electrostatic region. For retraction curves, the maximum number of discontinuities is introduced to constrain the number of regions of interest and prevent data analysis to be impaired by noise level. In case of retraction curves, the following issue occurs quite frequently: it arises that the force value is positive in decreasing regions (*i.e.* a number of data points corresponding to *z*_i_ ≥ 0 have a positive force value). In this abnormal case, fitting the data to the FJC model does not make sense and processing of the corresponding decreasing region is skipped since it does not carry any trustable information. In details, for any decreasing region yielded by the segmentation procedure, our processing is developed in such a way that it solely accounts for data points with a negative force value. With appropriate test-subroutine that detects the sign of the force in the decreasing regions, in case of ‘incorrect test’ (positive force value), we removed the corresponding decreasing interval and did not process the FJC fitting (the force curve is excluded from the analysis).

## Results and Discussion

The experimental part of this work has been carried out to illustrate the different steps of the data processing and address the consistency of the so-estimated physical parameters. This section is organized as follows. For both approach and retraction, we first illustrate the behavior of the proposed algorithm on a single force curve, and then, we provide results (2D maps representing the physical parameters of interest) obtained by processing the FVI. In particular, we strongly underline that the proposed algorithm is run on the whole FVI using a common parameter setting. We analyze the results and, in particular, discuss the accuracy of the obtained physical parameters, and compare their values with data reported in literature. Finally, we comment on the behavior and performance of the proposed algorithm and we qualitatively elaborate potential adaptations to other physical models.

### Data processing for the approach force curve

**Figure 4:**
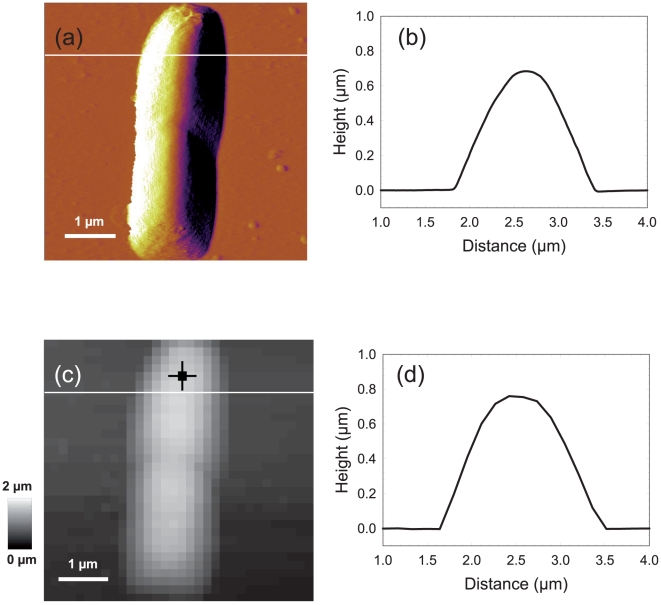
Comparison between deflection and Force Volume image resolution. Deflection image (a) and reconstructed height image (c) of *E. coli* cells in buffer solution (1 mM KNO_3_). Figures 4b and 4d correspond to the height profiles measured along the cross section of Figure 4a and Figure 4c as indicated therein by white lines.

The 512-by-512 pixel AFM image reported in Figure 4a reveals that *E. coli* E 2152 can be assimilated to 5 µm long rod-shaped cell being in the process of division. The horizontal line corresponds to the lateral cross section depicted in Figure 4b. The width (∼1.6 µm) of the cell was found to be larger than the height (∼0.7 µm), which is essentially caused by artifactual features connected to probe geometry. In order to perform the force volume measurements, a 5 µm×5 µm scan region was divided into a 32-by-32 grid. For each pixel on this grid, force curves were recorded upon approach of the probe toward the sample surface, using an applied force of 4 nN.

Prior to processing a force-volume image, it is necessary to set the values of the algorithm parameters. An important point is that these values are common to all force curves. First, a choice must be done regarding the polynomial degree *r* (for piecewise polynomial smoothing). We set 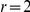 since approach force curves involve linear and convex regions which can be locally approximated by affine and quadratic polynomials. The choice for the number of iterations of the segmentation step, *i.e.* the number of discontinuity points, is critical. In the ‘Algorithm’ section, we introduced two alternative strategies where the number of iterations is maintained constant, or varies depending on data. Here, we use the first strategy since the number of discontinuity points is always limited and does not strongly vary from one curve to another. We observed that the approach curves are very well approximated with 6 discontinuity points. Figures 2a and 2b illustrate the segmentation results obtained for an approach curve measured on E2152 bacterial strain (marked pixel of Figure 4c). In details, the approach curve is first pre-processed by removing an affine baseline (typically, the first 500 data points upon approach of the tip). The pre-processed curve is displayed in Figure 2a and the force curve segmentation result (zoom in) is shown in Figure 2b. Figure 3a shows the exponential part of the same force curve while Figure 3b displays this force curve as a function of the indentation *δ* together with the fitting results in the Hertzian and Hooke regions. Both red line and arrow correspond to the positions 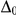 and 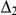 found by the optimization algorithm, the latter marking the transition between the Hertzian and Hooke regimes. The black line in between is the 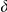-value 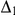 at *z* = *Z*_1_, marking the transition from the electrostatic to the Hertzian regime.

Let us now comment on the global results obtained when processing all force curves. A first way to assess the quality of the data processing consists in comparing the topographic reconstruction achieved *via* estimation of the *Z*_1_ values for all pixels, with the topography obtained *via* contact mode measurements. Figure 4b shows the cross section of the bacterial height in contact mode, Figure 4c displays the topographical reconstruction obtained from the processing of the FVI is given, and Figure 4d shows a cross section of this reconstruction. For each pixel, the height corresponds to the *Z*_1_ value, the 0-height being defined as the maximum value of *Z*_1_ over the whole image. Despite the low resolution of the FVI, the topographic reconstruction of the bacterium is in very good agreement with that of the high resolved AFM image of Figure 4a.

**Figure 5:**
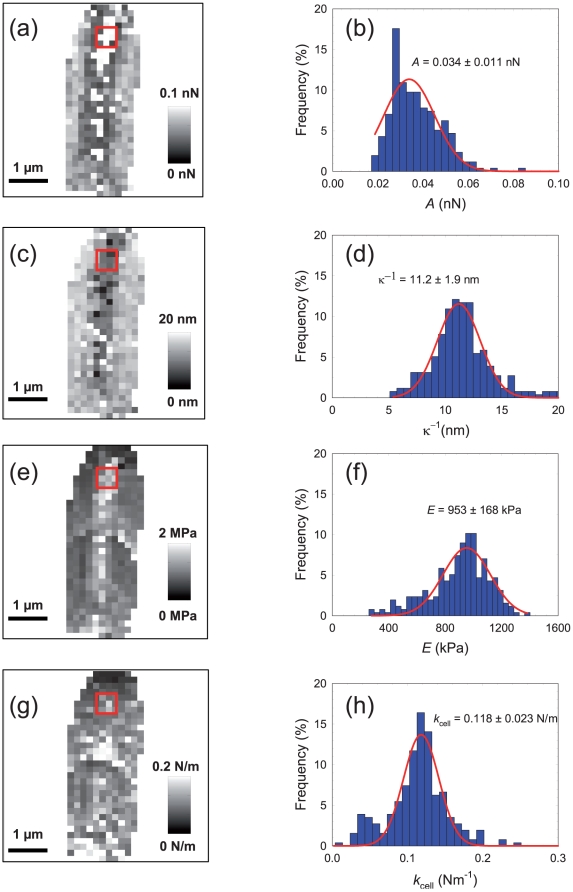
Physico-chemical properties of *E. coli* cells in buffer solution (1 mM KNO_3_). (a) 2D map of electrostatic prefactor *A* (*A*-range  = 0–0.1 nN). (b) Statistic distribution of electrostatic prefactor corresponding to 2D map in (a). (c) 2D map of the Debye length (κ^−1^-range  = 0–20 nm). (d) Statistic distribution of the Debye length corresponding to 2D map in (c). (e) 2D map of the Young modulus (*E*-range  = 0–2 MPa). (f) Statistic distribution of the Young modulus corresponding to 2D map in (e). (g) 2D map of the bacterial spring constant (*k*_cell_-range  = 0–0.2 N/m). (h) Statistic distribution of the bacterial spring constant corresponding to 2D map in (g).

Figure 5 displays the 2D maps of the obtained physico-chemical properties: pre-exponential factor *A* (panel a), Debye length *κ* ^−1^ (panel c), Young modulus *E* (panel e) and bacterial spring constant *k*_cell_ (panel g) extracted from the exponential and Hertz-Hooke regions of the measured force curves. The spatial distribution of the bacterial physical properties highlight that whatever the considered physical parameter, maximum values are located on the central part of the bacterium whereas the lower values are located on the edges. This spatial heterogeneity is mainly attributed to the effects of convolution between tip geometry and cylindrical shape of the bacteria when the latter is subjected to a normal force applied at the edge. Stated differently, the geometry of the contact zone bacterium/tip slightly differs according to whether force is applied at the center or at the edge of the cell. In order to minimize the effects of curvature of cell surface and increase the statistics of the obtained results, we performed additional force volume measurements within a grid of 16-by-16 points on the bacterial surface. The corresponding 500-by-500 nm^2^ area is marked by small red squares indicated in the images of Figure 5.

Figures 5b and 5d show the distributions of the parameters *A* and *κ* ^−1^ characterizing the electrostatic properties of the bacterial envelope as probed within the aforementioned red square region marked in Figures 5a, 5c, 5e and 5g. In particular, we note an excellent agreement of the most frequent value found for *κ* ^−1^ (11.2 nm) with that expected from theoretical eq (3) which yields *κ* ^−1^ = 9.8 nm for a 1 mM KNO_3_ electrolyte concentration as adopted in the experiment. The Young modulus was found to be 953±168 kPa (Figure 5f), in qualitative agreement with values obtained by AFM on yeast cells [94] (600±400 kPa), *Phaeodactylum tricornutum* morphotypes [20] (from ∼100 to ∼500 kPa), *Lactobacillus rhamnosus* (186±40 for wild type and 300±63 kPa for mutant), *Myxococcus Xanthus* (250±180 for wild type and 1340±660 for mutant) in MilliQ water. The value obtained for the bacterial spring constant (0.118±0.024 N/m in Figure 5h) is in line with that estimated for *Staphylococcus epidermidis* in water [95] (0.24±0.01 N/m) and for filamentous fungal *hyphae* in PBS buffer [96] (0.29 - 0.17 N/m depending on osmolarity). Schar-Zammaretti *et al.* [97] reported smaller values: 0.05 N/m for *L. crispatus* and 0.03 N/m for *L. helveticus* in 10 mM KH_2_PO_4_ buffer at pH 7. Finally, we indicate that the results obtained here are very consistent with those reported elsewhere for the same bacteria and obtained according to manual data processing [43].

### Data processing for the retraction force curve

The elastic and mechanical properties of microbial surface macromolecules of *P. fluorescens* as well as their spatial distribution on the surface were probed *via* single-molecule force spectroscopy (SMFS). The 5 µm×5 µm scan region was divided into 32-by-32 pixels similar to the analysis of the approach force curves previously detailed. For each pixel, the modified tip was brought into contact with the biopolymer molecules located at the bacterial surface. The force curves were then recorded upon retraction of the probe tip from the surface of the sample. Figures 2c and 3d show typical data for the force *versus* sample to tip distance together with theoretical reconstruction based on FJC model (eq. (6)). Let us recall that the first 100 nm for each retraction curves are not processed because this part includes ill-defined dependence of the force versus separation curve that possibly corresponds to the concomitant occurrence of intricate physical phenomena like binding/removal of certain macromolecules on/from the tip or macromolecule rearrangement on the tip surface.

In the segmentation algorithm, both stopping conditions detailed in the ‘Algorithm section’ are combined. Fixing a maximal value for the number of discontinuity points ensures that the algorithm runs within a tractable computation time (30 seconds at most) while setting a threshold value for the mean squared error 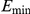 is required because the number of regions of interest may strongly differ from one force curve (corresponding to a given pixel in the force-volume image) to another. The 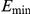 value is taken identical for all curves. We set 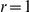 and 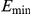 to the empirical variance of the noise estimated from the flat part of the force curve. The segmentation of the retraction curve is illustrated on Figure 2d. Similar to pre-processing of the approach curve, an affine baseline is subtracted from the raw data. Here, the maximal number (20) of discontinuities is reached. For other force curves constituting the force-volume image, the stopping condition relying on 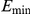 criterion is met and less than 20 discontinuities are detected.

**Figure 6:**
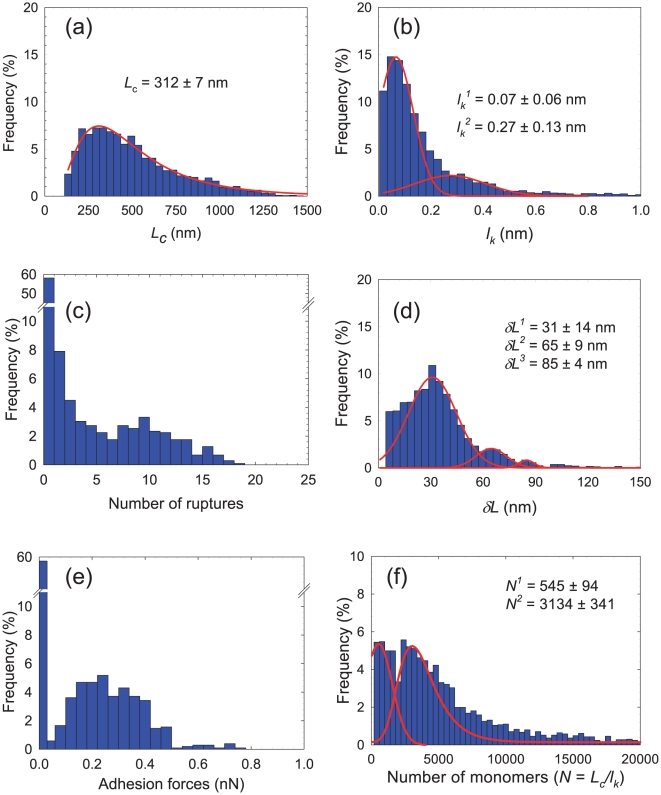
Physico-chemical properties of *P. fluorescens* exopolymers. (a) Statistic distribution of the contour length. (b) Statistic distribution of the Kuhn length. (c) Statistic distribution of the number of ruptures (number of regions of interest +1). (d) Statistic distribution of the distance between two consecutive adhesion force ruptures. (e) Statistic distribution of the adhesion force (amplitude of the last adhesive event). (f) Statistic distribution of the number of monomers constituting the polysaccharidic chains (*N* = *L_c_*/*l_k_*).

**Figure 7:**
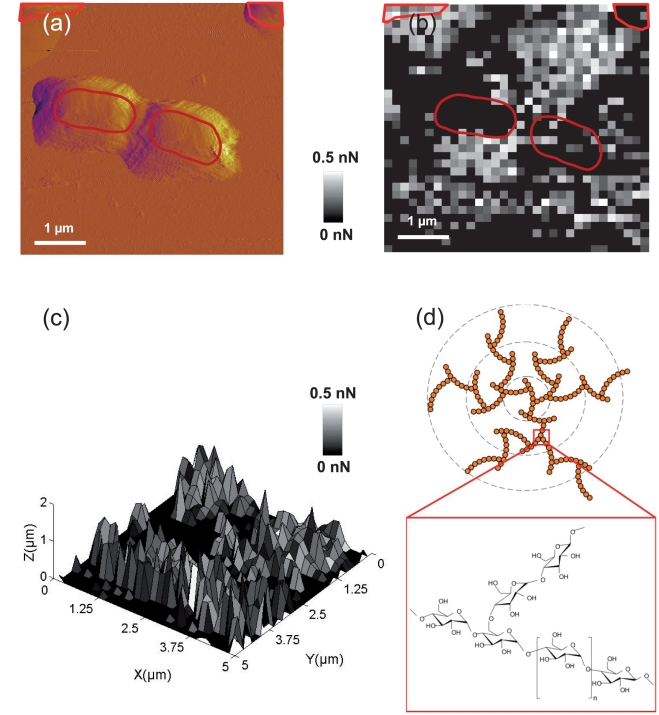
Maps of adhesive properties of *P. fluorescens*, and glycogene structure. (a) Deflection image of *Pseudomonas fluorescens* taken before FVI experiment, the red zones correspond to bacterial cells location. (b) Adhesion force map (*F*-range  = 0–0.5 nN) for the last adhesion force rupture measured on bacteria located on the surface as indicated in panel a. (c) Three dimensional map of adhesive properties of *P. fluorescens* combining the last adhesion force and last rupture distance corresponding to uncoiled exopolymers. (d) Fractal structure of the poly glycogene and chemical formula.

Figures 6a and 6b show the frequency distribution (over the whole FVI) of both fitting parameters involved in the FJC model: the contour length *L_c_* and the Kuhn length *l_k_*. The total contour length of the macromolecule found at the bacterial surface ranges from 100 nm to 1250 nm with a most frequent value of *L_c_*≈312 nm. The histogram for the distribution of the Kuhn length *l_k_* shows two pronounced maxima at *l_k_*≈0.07 nm±0.06 nm and 0.27±0.13 nm. The first value is very close to 0.065 nm, which corresponds to the elongation of a single sugar molecule following a conformational change of a C_5_-C_6_ bond position. The second value is in quantitative agreement with the result of Camesano *et al.* [70] (0.23±0.11 nm for polysaccharides on the surface of *P. putida* in 0.1 M KCl). Furthermore, previous studies evidenced a Kuhn length of about 1.2 nm to 1.4 nm for *Lactobacillus rhamnosus* EPS's [21] which consist in the repetition of 6 sugars [98], [99]. This latter result again indicates that the Kuhn length 0.27 nm obtained for *P. fluorescens* probably corresponds to the size of a glucose molecule. This is confirmed by independent FTIR measurements (data not shown) which demonstrate that *P. fluorescens* produces poly glycogene, strictly composed of glucose units (paper in preparation). Figure 6c shows that one to twenty uncoiling/rupture events can be detected over the whole retraction curves forming the FVI. The *z*-distance between two consecutive adhesion events *δL* (see Figure 1d for the definition of *δL*) is related to the length between two branches in the poly glycogen chain. The histogram representing the frequency distribution in δ*L* over the whole FVI (Figure 6d) yields three pronounced maxima at 31±14 nm, 65±9 nm and 85±4 nm. The observed periodical length *l_p_* of ∼30 nm can be attributed to the shortest distance between two branches within a given poly glycogen chain, since unfolding may occur simultaneously on the various parts of the macromolecule. In addition, the histogram (Figure 6f) which depicts the distribution of the number of monomers (*N* = *L_c_*/*l_k_*), exhibits two peaks. The first peak accounts for about 25% of the polysaccharidic chains with 545 monomers while the second peak corresponds to about 70% of the polysaccharidic chains with 3134 monomers. This result suggests the presence of both short and very long polysaccharidic chains, which are in line with previous studies carried out on the ramified structure of glycogen (see Figure 7d). Melendez-Hevia *et al.* [100], [101] optimized several structural parameters of glycogen to achieve efficient fuel molecules for energy storage in cells (optimization of cell metabolism). Their results show that this molecule is formed by concentric poly glycogene fractal chains with 12 branches (see Figure 7d). A rough estimation from our data suggests *L_c_*/*l_p_*≈10 branches. The discrepancy may originate from the fact that the fuel storage is not critically governed by the structure of poly glycogene outside the cell. The statistical distribution of adhesion forces, shown in Figure 6e, evidenced that polysaccharides are absent or not detectable from more than 60% of the total FVI area. Furthermore, we attribute the adhesion forces in the range 0.1–0.5 nN to the simultaneous detection of two to ten macromolecules, as judged from independent calibration SMFS measurements performed on glucosamine grafted-gold surface (see File S1) and literature [74], [102]. The distribution of adhesion force is located around the bacterial cells (Figure 7a and 7b), thereby suggesting that *P. fluorescens* excretes EPS outside the cell wall.

Figure 7a shows the deflection image obtained in contact mode for *P. fluorescens*. In order to quantitatively clarify the distribution of the number of macromolecules on the surface, we considered the adhesion force corresponding to the last peak detected in the retraction curves. The amplitude of the adhesion force in this case is proportional to the number of macromolecules simultaneously detached from the tip. The corresponding 2D map is presented in Figure 7b. To better visualize differences in polysaccharide properties, 3D-map was constructed by combining last rupture distances (*z* level) and adhesion forces (colors) measured at every (*x*, *y*) location (see Figure 7c). From direct comparison of Figures 7a, 7b and 7c, it turns out that the cell is not covered entirely by polysaccharides which are distributed mostly around the cell. To conclude this part, we mention that not only the results are in good agreement with those in AFM literature [21], [89], [103], [104], but they are also in line with data obtained with other techniques such as FTIR. In addition, to the best of our knowledge, these results provide the first complete large scale physico-chemical analysis (*i.e.* on/outside the bacterial cell) of such macromolecules on living organisms.

### Discussion on the performance of the algorithm for force curve analysis

In the previous parts, the focus was mainly given to the relevance of the estimated physical parameters. We now evaluate the performance of our proposed algorithm from a signal processing perspective. We also discuss some possible extensions of the algorithm.

A first aspect is the robustness of the analysis with respect to the design parameters (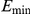, number of discontinuities, initial conditions, *etc*). For the retraction curve, the main tuning parameter of the segmentation algorithm is 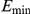 which controls quality of the fit. Basically, if 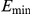 is low, the number of detected discontinuities tends to a large value. This has motivated the introduction of a maximum number of discontinuities as additional parameter. For all experimental data, the value of 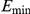 was set to the empirical noise variance estimated on the flat portion of the curves. A number of experiments have been performed to assess the influence of the number of discontinuities. It appears that, as long as the number of discontinuities is chosen large enough, the segmentation procedure is not very sensitive to this parameter. In case of Figure 6c, the maximal number of discontinuities is only reached a few times. For the other force curves, the maximal number is not reached, meaning that the algorithm stopping was actually controlled by 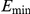. As a great attention has been paid to the choice and set-up of these parameters, it is worth noticing that all 1024 force curves of the reported FVI were processed using the same tuning. The procedure is pretty robust as an analysis of the curves starting with the key physical parameter values departing from those required for fitting, always ends up with the same convergent results. The effectiveness of the algorithm can also be assessed by looking at square error maps as depicted in Figure S3. These maps clearly show low and homogeneous fitting errors, all over the spatial domain corresponding to the bacterium.

A second aspect is the robustness of data processing in relation to the choice of the physical model. This is a question admitting no definitive answer and we may just give some insights for clarification. To be specific, let us consider the piecewise parametric model (electrostatic, Hertzian, Hookian). Each of the 3 types of interaction only gives an approximate representation of the ‘truth’ but has the advantage of being numerically tractable and well interpretable. A fully satisfactory model should indeed be able to recover the full interaction force curve on the basis of a single expression that would, among other features, adequately represent the highly non-linear transition from one interaction regime to another. Such model does not exist and we are then left with our segmentation procedure that goes in pair with the splitting of the force curve into distinct interaction regimes. Though surely improvable, this method is efficient and provides physically relevant values of parameters (see *e.g*. values of Debye length as compared to expected theoretical magnitude). Another question is the relevance of the piecewise assumption. As mentioned above, this is still a rough approximation of truth, but it has the advantage of yielding numerically tractable and well interpretable results. In that respect, the method is robust, or at least much more robust than a method that would employ more sophisticated models yielding less stable and interpretable results. This is typically the kind of problems which has to be faced when fitting data to FJC+ or WLC+ models, problems which are typically ill-conditioned. It is beyond the scope of the current study to discuss this in details but we believe that the stable fitting of data to this kind of ill-conditioned models is the most challenging point to be addressed for extending the proposed approach to more complex models.

The FVI processing was always performed after its whole acquisition (20 to 30 minutes) resulting in an off-line procedure. However, as the processing of a given force curve is independent of that for another (except for some initialization), and because the time for processing each approach curve is fast (typically, 1 to 2 seconds), it seems possible to perform the processing of one force curve while acquiring the next one, resulting in a quasi on-line processing. The time for processing each retraction curve is significantly larger (about 24 seconds on average) and therefore a quasi on-line processing of such data would surely benefit from an implementation of the algorithm in a compiled language (*e.g*., in C).

Although there is no strong memory requirement for the proposed algorithm to run correctly (only one force curve must be stored at a time), it could be relevant to reduce the overall memory storage by performing data compression for appropriate storage of complete FVI. Rather than using standard data compression method, we believe that a fruitful option could be the development of data compression tools specifically adapted to the very nature of the measured force curves. In that respect, our force curve segmentation algorithm may be viewed as a *signal compression* tool since it allows for coding the original measured signal with help of a few critical key parameter values (the number of discontinuity points).

### Conclusion

We proposed original automatic algorithms for processing approach and retraction force curves of FVI's. Their common feature is the accurate detection of key regions of interest where available force physical models may be applied for quantitative interpretation of the data. We automatically processed several force-volume images by running the algorithms for analyzing all force curves recorded at the pixels constituting the image. This processing allows us to provide statistical and spatial distributions of electrostatic and nanomechanical properties of biological samples together with characteristic features pertaining to their outer polymeric materials (specific adhesion). All the procedures are gathered in a software allowing for the automatic processing of a FVI.

The proposed algorithm was tested on real data obtained with AFM. The approach experiments were performed on *E. coli* bacterium without surface appendage. For the retraction case, we investigated surface properties of *P. fluorescens* which produces exopolysaccharides (EPS). For the approach force curves, the detected discontinuities are associated to critical points marking the transition between regions where electrostatic, non-linear (Hertz) and linear (Hooke) contact mechanical interactions take place. For retraction curves, the detected points are related either to unfolding or to detachment of the EPS macromolecules from the AFM tip.

The data were then fitted to theoretical expressions for obtaining relevant physical parameters pertaining to *E. coli* bacterial cell wall: the Debye length, Young modulus (stiffness of bacterial envelope) and turgor pressure (inner osmotic pressure). In addition we analyzed detailed structural, conformational and mechanical properties of exopolysaccharides from *P. fluorescens* cell wall, *i.e.* Kuhn length, contour length and parameters describing the the fractal structure of the macromolecule [101], [105], [106]. Inspection of the obtained spatial distribution clearly shows an accumulation of polysaccharides around *P. fluorescens* and not only over the cell wall. The results are in quantitative agreement with data from literature. Several benefits in using the proposed algorithm need to be stressed. For the first time, electrostatic properties of bacterial envelope without surface appendage are unambiguously extracted. Detection of the transition points between different force regimes increases the reliability of the obtained results for the approach experiments and makes it possible to perform automatic fitting of retraction curves without manual selection of the regions of interests.

An interesting perspective of this work consists in the concomitant analysis of several force curves while the present algorithm processes all measured force curve in an independent manner. It is indeed of interest to take into account the fact that some of the physical parameters (*e.g.* the topography of the sample) do not strongly vary from a given pixel to its neighboring ones. An extended tool could then estimate the physical parameters relative to a pixel based not only on the corresponding force curve but also on the force curves recorded at neighboring locations of the sample surface. This would surely allow for finer parameter estimation.

Further extension could easily include more complex and detailed models developed *e.g.* for elastic deformation [41], [65] (see details in File S1). Our numerical strategy could further surely be extended to the analysis of data obtained on other biological systems (human cells, micro-algae) and other types of interactions (in particular short range specific interactions upon approach) providing that corresponding physical models are available. For the sake of illustration, the physico-chemical characterization of animal cell biomacromolecules or the evaluation of polymer layer density on eukaryotic cells (by molecular stretching) could be automatically performed adopting the strategy reported here. Finally, we mention that our procedure may be used for the estimation of viscoelastic properties of cells according to effective modeling approach based on Hertz model. Along this line, one determines the dependence of the Young modulus on the speed according to which the tip approaches the tip, or one evaluates the ‘slopes’ of trace and retrace for a given tip speed. The amount of force curves to be automatically processed would then be significantly larger than that typically considered in this work devoted to analysis of static (steady-state) measurements. Extension of our algorithm to the analysis of full dynamic measurements (approach and retraction force curves at various tip speeds [107], [108], determination of mechanical relaxation time) is also thinkable. Such extended algorithm is possible on the premise that the physics of surface dynamics is well-understood (in order to achieve appropriate fitting), and that the time for processing is adequately and carefully optimized (on-line processing, coupled processing of different force curves, analysis of data processing speed in relation to speed of data acquisition, etc).

## Supporting Information

Figure S1 **Determination of the turgor pressure.** (a) Dependence of the bacterial spring constant on the inner bacterial pressure for 4 different shear moduli *λ* (respectively 0.100, 0.114, 0.200 and 0.400 N/m). The dashed horizontal line corresponds to the *k*_cell_ value measured by AFM. (b) 2D map of turgor pressure (*P*_0_-range  = 0–300 kPa). (b) Statistic distribution of the inner bacterial pressure corresponding to 2D map in (b).
(EPS)

Figure S2
**Retraction force curve analysis.** (a) Segmentation results. The polynomial degree is set to *r* = 2 leading to a piecewise quadratic approximation of the original signal. The threshold value 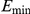 for the mean squared error is set to the empirical noise variance, and 20 discontinuities at most are detected. For the displayed retraction curve, 20 iterations were performed leading to 20 discontinuities. (b) Retraction curve: detection of the regions of interest (decreasing regions of the piecewise affine signal). (c) Fitting of the data to the FJC model in each region of interest. No fitting is performed in the first 100 nm after the contact point 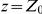. For each region, the extrapolation of the FJC model outside the current region of interest is shown in green.(EPS)

Figure S3 **Maps of fitting error performed on** ***E. coli*** **cell for electrostatic and Hertz-Hooke models.** (a) Fitting error map for the electrostatic model (eq (7)). (b) Fitting error map for the Hertz-Hooke model (eq (9)). The bacterial cell contour is depicted by the red line.
(EPS)

Figure S4 **Single-molecule force experiments and tip deactivation on model surface.** (a) Statistic distribution of adhesion force measured on glucose coated-gold surface. (b) Statistic distribution of adhesion force measured on bacterial cells after injection of 2 mL of glucose solution at 100 mM.
(EPS)

Figure S5 **Statistic distribution of adhesion force measured on PEI-coated glass surface.**
(EPS)

Figure S6 **Schematic representation of different polymer models.** FJC and WLC taken from [86]. Typical monomers constituting exopolysaccharides (left) and proteins (right).
(EPS)

File S1 (DOC)
