# Supplementary material for: Automated Force Volume Image Processing for Biological Samples
Source: PLoS One. 2011 Apr 29;6(4):e18887. doi: 10.1371/journal.pone.0018887 (PMC3084721; doi:10.1371/journal.pone.0018887)
Supplement: File S1 — (DOC) [file pone.0018887.s007.doc]

## **Supporting Information S1**

**Turgor pressure calculation.**

The following expression for the cell spring constant was obtained by minimizing the elastic free energy of the bacteria [109]:

$k_{cell}=\alpha P_{0}R\phi\left(\frac{r_{c}}{d_{c}}\right),$ (1)

where $\alpha=3\pi/2$ for a cylindrical bacterium, *R* is the radius of the bacterium (*R* is equal to 0.35 µm for the E2152 bacteria investigated in our study), *P_0_* is the pressure difference across the bacterial envelope (the turgor pressure), *r_c_* is the radius of the probe tip and *d_c_* is a cut-off distance for the range of the elastic deformation on the bacterial surface. For cylindrical bacteria, we have:

$d_{c}=\sqrt{\frac{PR^{3}}{\lambda-PR}},$ (2)

where *λ* is the laternal modulus of compression. The function $\phi$ involved in eq (13) reads as:

$\phi(x)=x\frac{K_{1}(x)}{K_{0}(x)},$ (3)

where *K_n_(x)* is the modified Bessel function of order *n*. The laternal modulus of compression *λ* was previously measured by micropipette aspiration method for guinea pig cochlear outer hair cells (15.4 ± 3.3 mN/m) [110] and by optical tweezers micromanipulation of small silica beads bound to the membrane for human red blood cells (2.5 ± 0.4 mN/m) [111]. Boulbitch [109] used *λ* = 0.1 N/m, but did not specify the origin of this value. Arnoldi *et al*. [112] estimated *λ* ~ 0.1 N/m for gram negative *Magnetospirillum gryphiswaldense* from the Young modulus and the thickness of the bacterial envelope. For *E. coli* we did not find any data for *λ* in the literature. The turgor pressure for the given spring constant *k_cell_* (eq (1)) was found by searching for the minimal value of laternal modulus of compression *λ* which satisfies eq (1). Figure S1a shows the dependence of spring constant *k_cell_* versus the turgor pressure (*P_0_*) for different values of *λ* = 0.100, 0.114, 0.200 and 0.400 N/m. It is clear that the parametric curves adequately reproduce the experimentally determined *k_cell_* = 0.120 N/m for λ ≥ 0.114 N/m. Realizing that the laternal modulus of compression *λ* is a measure of the surface free energy of the bacterial envelope, we may postulate on the basis of thermodynamic arguments that a good approximation for *λ* corresponds to the minimum value *λ_min_* 0.114 N/m (*e.g.* minimum bacterial surface energy). The turgor pressure was found to be 250 ± 49 kPa (Figure S1), which is in quantitative agreement with AFM data on gram negative *Pseudomonas aeruginosa* [86] (150 – 400 kPa) and *Magnetospirillum gryphiswaldense* [112] (85 – 150 kPa) in distilled water. Koch [113] calculated turgor pressure of gram negative *Ancylobacter aquaticus* (187 ± 28.3 kPa) as the difference between the collapse pressure of the cell vesicles with and without sucrose present in the medium.

# Other physical models relevant for retraction force curve analysis

In the extended FJC+ model, the polymer segments are assimilated to elastic springs of segment elasticity denotes as *k_s_*. The expression for the macromolecule extension then reads as [87,88]:

$z_{\text{FJC}+}\left(F\right)=-L_{c}\left[\coth\left(\frac{Fl_{k}}{k_{\text{b}}T}\right)-\frac{k_{\text{b}}T}{Fl_{k}}\right]\left(1+\frac{F}{k_{s}L_{c}}\right)$. (4)

where *F* is the pulling force, $L_{c}$ is the total contour length of the macromolecule and $k_{\text{b}}$ is the Boltzmann constant.

Within the framework of the WLC model, it is assumed that the macromolecule consists of an irregular curved filament, which is linear on the scale of the persistance length *l_p_*. The pulling force *F* may then be expressed as a function of the macromolecule extension *via* the expression [87,88]:

$F_{\text{WLC}}\left(z\right)=-\frac{k_{B}T}{l_{p}}\left[\frac{1}{4}\left(1-\frac{z}{L_{c}}\right)^{-2}+\frac{z}{L_{c}}-\frac{1}{4}\right],$ (5)

In the extended WLC+ model, a specific stiffness of the macromolecule is introduced. The expression for the pulling force then becomes:

$F_{\text{WLC}+}\left(z\right)=-\frac{k_{B}T}{l_{p}}\left[\frac{1}{4}\left(1-\frac{z}{L_{c}}+\frac{F}{\varphi}\right)^{-2}+\frac{z}{L_{c}}-\frac{F}{\varphi}-\frac{1}{4}\right],$ (6)

where $\varphi$ is the specific stiffness of the macromolecule, *l_p_* is the persistence length. In Fig. S6, we give schematic representations of polysaccharidic and polypeptidic macromolecules for which FJC and WLC modeling best apply, respectively.

**Algorithm extension for force curve analysis**

We now discuss further issues to be considered for potential extensions of the FVI processing algorithm presented in the main text. Although the algorithm has been described for specific parametric models (electrostatic interaction, Hertz and Hooke interaction, FJC model), it should be recognized that the fitting step can be extended to other models. In the approach case, other Hertz models could be used within the same optimization framework. For instance, when the shape of the AFM tip is modeled as a sphere, the Hertz model differs and reads as *F*_Hertz_(*δ*) = *B’δ*^3/2^ instead of *F*_Hertz_(*δ*) = *Bδ*^2^ [65]. A more precise expression was proposed by Dimitriadis *et al.* [41]. An extension of the present work could naturally include these more complex models in the fitting algorithm.

In the retraction case, several adaptations of the proposed algorithm are possible. First, let us notice that the fitting of experimental data to the FJC model in each region of interest $R_{j}$ leads to the estimation of the persistence length $l_{k}^{j}$ and the contour length $L_{c}^{j}=L_{c}(l_{k}^{j})$, the number of monomers reading as $N_{j}=L_{c}^{j}/l_{k}^{j}$. Clearly, this quantity is generally not an integer. If one desires to impose the constrain that this number is an integer, one needs to perform a small post-processing for replacing $N_{j}$ by its closest integer, and subsequently updating the values of $l_{k}^{j}$ and $L_{c}^{j} = N_{j} l_{k}^{j}$. This post-processing takes the form of a 1D local optimization problem. We observed that this post-processing leads to very minor changes of the $l_{k}^{j}$ and $L_{c}^{j}$ values and to a negligible increase in the approximation error.

Similar to the approach case, we considered the adaptation of the algorithm to other retraction models. We performed the fitting of data according to the WLC model [114,115,116] by designing an appropriate least-squares cost function. Contrary to the cost function defined in the manuscript by eq (10), the squared error now relies on the difference between the observed *F_i_* values and their approximation based on the WLC model the form *F_WLC_*(*z*). The WLC parameter estimation can be performed successfully under appropriate specification of mild constraints on the parameter values and realistic setting of their initial guesses. This kind of model is usually adapted to protein and polypeptides. For polysaccharide macromolecules, the generally accepted model is FJC+ which takes into account monomer elasticity. Using the FJC+ model rather than FJC enables us to improve the quality of fit, *i.e.* to decrease the error between the experimental data and their approximation in each region of interest. This improvement is related to the additional parameter *k_s_* involved in the FJC+ model. However, when subsequently processing the curves of a given FVI, we observed two kinds of behavior. In the first case, the obtained FJC+ parameters are very realistic and in coherence with their expected values. On the contrary, numerical issues occur for a number of pixels where the cost function to be minimized (corresponding to the approximation error in the given region of interest) takes a degenerate form. Indeed the minimizer of the cost function may be located inside a flat valley, making the fitting problem very ill-conditioned. In other words, a large range of values for *l_k_*, *L_c_* and *k_s_* leads to the same approximation error. In such case, it is mandatory to impose additional constraints (*e.g.* forbidding some ranges of values for *l_k_*, *L_c_* or *k_s_*) to cope with the numerical optimization issues and obtain reliable and realistic estimation of the relevant physical parameters.

**Verification of the functionalized AFM-tips.**

SMFS experiments require the use of functionalized silicon nitride AFM-tips. For the current study, AFM-tips were functionalized with *Concanavalin* A (ConA) known for binding specifically glucose and mannose molecules. Prior to the analysis and detection of polysaccharides on the bacterial surface, we verified the quality of functionalization and the binding specificity of the functionalized AFM-tips. For this purpose, force curves recorded on glucose coated-gold surface (Figure S4a) evidenced 90% of adhesive events and a Gaussian distribution for a specific binding force of 0.058 ± 0.017 nN between ConA and glucose. After injection of few ml of a solution of glucose (100 mM), we observed an important decrease of the adhesive events (17%) reflecting desactivation of the functionalized AFM-tip by free glucose in solution (Figure S4b). In addition, before each experiment, we verified that ConA tips did not bind specifically to the PEI-coated glass (see Fig S5). For this purpose, statistic analysis of adhesion highlighted about 10% of adhesive events between ConA and PEI.
